# PD52 Prevalence Of Low Back Pain In The Adult Population Living In Brazil: A Systematic Review And Meta-Analysis

**DOI:** 10.1017/S0266462325101840

**Published:** 2025-12-29

**Authors:** Maria Augusta de Araujo Mota, Yara Marques, Alne Toledo, Rodrigo Carregaro

## Abstract

**Introduction:**

Although several studies have explored the prevalence of low back pain (LBP) in Brazilian individuals, no meta-analysis has yet been carried out to comprehensively estimate epidemiological aspects, such as the point, period, and lifetime prevalence of LBP in this population. The aim of this systematic review and meta-analysis was to investigate the one-off, annual, and lifetime prevalence of LBP in the Brazilian population.

**Methods:**

Systematic searches were carried out in the following databases, with no restriction on publication date: MEDLINE (Ovid), Embase, Web of Science, Scientific Electronic Library Online, and LILACS. Google Scholar was also checked. The reference lists of included studies were also screened for potentially relevant studies. Prevalence rates per period were combined and calculated.

**Results:**

Sixty-seven studies were included in the review. Eight of these studies (11.8%) were of higher methodological quality. The pooled prevalence of LBP was 23 percent (95% confidence interval: 11, 43) based on a random-effects model. Heterogeneity was extremely high (I²=99.5%; p<0.0001), indicating significant variability between studies. Each study contributed equally to the analysis (approximately 12.5% weight).

**Conclusions:**

LBP prevalence in Brazil exceeds global averages, affecting a significant portion of the population. This study highlighted the need for policies focused on LBP prevention and management, to improve health outcomes for Brazilians and potentially benefit a large global population.

